# Response to the letter to the editor: Methodological considerations in long-term diabetes mortality studies

**DOI:** 10.20945/2359-4292-2026-0097

**Published:** 2026-08-23

**Authors:** Lucas Casagrande Passoni Lopes, Marina dos Santos de Carvalho Pinto, Mauro Wieczorek, Carlos Antonio Negrato

**Affiliations:** 1 Universidade de São Paulo, Faculdade de Medicina de Bauru, Bauru, SP, Brasil

Dear Editor,

We would like to thank Chris Munayco Carhuamaca for her letter entitled “Methodological considerations in long-term diabetes mortality studies” (^1^) for her careful appreciation of our article “Death cause and age at death among individuals with diabetes: a 40-year retrospective Brazilian study” (^2^) and for their pertinent considerations presented. We believe that the observations highlighted contribute to deepening the scientific discussion on mortality studies in diabetes mellitus (DM) and offer a valuable opportunity to contextualize our findings.

We agree that analyses spanning for long decades are subject to the influence of substantial changes in the clinical management of DM, the availability of therapies, diagnostic criteria, and health information systems. Such transformations can introduce effects related to the observation period and should be considered in the interpretation of long-term longitudinal studies. As previously discussed, time-related biases represent a relevant methodological challenge in observational studies and can influence epidemiological estimates when important historical changes occur over the course of follow-up (^3^).

We also acknowledge the inherent limitations of using death certificates as a primary source for determining causes of death. Although they constitute one of the main tools for population-based mortality studies, their quality depends on multiple factors, including the correct identification of the causal chain of events leading to death and the proper completion of the death certificate by the certifying physician. International evidence demonstrates that errors and inconsistencies in the certification of causes of death remain frequent, and may impact the quality of mortality statistics (^4^). In this context, we agree that the underreporting of DM in death certificates represents a significant limitation. As cited, a previous study by our group identified a high frequency of omission of DM as a cause of death, particularly among individuals with type 2 DM and men (^5^). This aspect was discussed in the limitations section of the original article, as we recognize its potential to introduce classification bias and influence estimates related to mortality attributed to DM. On the other hand, we understand that these limitations reflect challenges widely recognized in the literature and inherent in the use of secondary mortality databases (^5^).

We agree that future investigations could benefit from analyses stratified by calendar period, sensitivity assessments for potential misclassification errors, and the incorporation of additional clinical information when available. We thank the author again for the constructive contribution and the opportunity to broaden the debate on the interpretation of mortality studies in DM.

## Data Availability

datasets related to this article will be available upon request to the corresponding author.
